# The Co-Creation of a Psychosocial Support Website for Advanced Cancer Patients Obtaining a Long-Term Response to Immunotherapy or Targeted Therapy

**DOI:** 10.3390/curroncol32050284

**Published:** 2025-05-19

**Authors:** Laura C. Zwanenburg, Marije L. van der Lee, José J. Koldenhof, Janneke van der Stap, Karijn P. M. Suijkerbuijk, Melanie P. J. Schellekens

**Affiliations:** 1 Center of Research on Psychological Disorders and Somatic Diseases, Department of Medical and Clinical Psychology, Tilburg University School of Social and Behavioral Sciences, 5037DB Tilburg, The Netherlands; lzwanenburg@hdi.nl (L.C.Z.); marije.vdrlee@tilburguniversity.edu (M.L.v.d.L.); 2 Department of Scientific Research, Helen Dowling Institute, Centre for Psycho-Oncology, 3723MB Bilthoven, The Netherlands; 3 Department of Medical Oncology, University Medical Centre Utrecht, Utrecht University, 3582CX Utrecht, The Netherlands; j.koldenhof@umcutrecht.nl (J.J.K.); k.suijkerbuijk@umcutrecht.nl (K.P.M.S.); 4 Department of Respiratory Diseases, University Medical Centre Utrecht, Utrecht University, 3582CX Utrecht, The Netherlands; j.vanderstap@umcutrecht.nl

**Keywords:** neoplasm metastasis, immunotherapy, molecular targeted therapy, psychosocial support systems, psychological wellbeing, e-health

## Abstract

Due to new treatment options, the number of patients living longer with advanced cancer is rapidly growing. While this is promising, many long-term responders (LTRs) face difficulties adapting to life with cancer due to persistent uncertainty, feeling misunderstood, and insufficient tools to navigate their “new normal”. Using the Person-Based Approach, this study developed and evaluated a website in co-creation with LTRs, healthcare professionals, and service providers, offering evidence-based information and tools for LTRs. We identified the key issues (i.e., living with uncertainty, relationships with close others, mourning losses, and adapting to life with cancer) and established the website’s main goals: acknowledging and normalizing emotions, difficulties, and challenges LTRs face and providing tailored information and practical tools. The prototype was improved through repeated feedback from a user panel (*n* = 9). In the evaluation phase (*n* = 43), 68% of participants rated the website’s usability as good or excellent. Interview data indicated that participants experienced recognition through portrait videos and quotes, valued the psycho-education via written text and (animated) videos, and made use of the practical tools (e.g. conversation aid), confirming that the main goals were achieved. Approximately 90% of participants indicated they would recommend the website to other LTRs. The Dutch website—Doorlevenmetkanker (i.e., continuing life with cancer) was officially launched in March 2025 in the Netherlands.

## 1. Introduction

Long-term responders (LTRs) are patients with advanced (i.e., unresectable metastatic) cancer who obtain durable survival rates due to effective treatment with new medical therapies such as immunotherapy or targeted therapy [1,2]. While it is good news that treatment is effective in prolonging life, living long-term with an uncertain prognosis comes with challenges. Approximately half of these patients report heightened levels of distress or fear of worsening of their disease [3,4]. LTRs often feel misunderstood by their social environment because most people live under the impression that individuals either recover or die from cancer rather than living with advanced cancer for an extended period [1]. Due to their uncertain life perspective, LTRs go back and forth between feeling like a patient or feeling healthy. The persistent uncertainty about the potential recurrence or progression of cancer makes it difficult for LTRs to make future plans. Consequently, many LTRs struggle to adapt to life with advanced cancer [1] and report multiple unmet needs [2,5,6].

To help them adapt, there is a need for support that is specifically tailored to LTRs because they find no recognition in the existing information for curative or palliative-treated patients [1]. As a result, one of the most pressing unmet needs reported by LTRs is the need for more tailored information [2,5,6]. We would like to offer LTRs easily accessible and evidence-based information and practical tools to acknowledge that living with cancer comes with many challenges and to help them manage the continuing stressors and uncertainty they are facing. Providing readily accessible support via a website can potentially prevent psychological problems and reduce the need for psychological care. As the LTR group is expected to grow in the coming years [7], placing additional strain on the healthcare system, a website designed for LTRs to use independently–requiring no support from healthcare providers–could help alleviate this burden.

**Figure 1:**
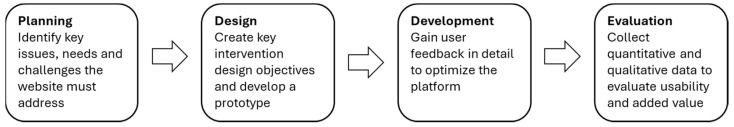
Person-Based Approach.

There is widespread consensus in the eHealth research community that eliciting and addressing the needs and perspectives of the intended user is a vital part of good intervention development to ensure (at a minimum) that the website is usable and engaging. This has been confirmed by previous studies in which web-based interventions were developed in co-creation with (advanced) cancer patients and healthcare professionals, showing that the co-creation process provided unique insight into the requirements of the intervention, offering a strong foundation to aid further development (e.g., [8,9,10]). In turn, patients positively evaluate these co-created interventions, indicating good usability, strong functionality, and added value. Since fully anticipating user priorities and needs is challenging, the Person-Based Approach by Yardley and colleagues provides a structured way to improve user experience and strengthen theory- and evidence-based intervention developments (see Figure 1) [11]. It involves qualitative research with LTRs and psychologists carried out at every stage of intervention development. Insights from this process are used to anticipate and interpret website usage and outcomes and, most importantly, to modify the website to make it more feasible and relevant to LTRs. The aim of this study is to develop a psychosocial support website for long-term responders in co-creation with patients, psychologists, and service providers and evaluate the feasibility and usability of this website using the Person-Based Approach [11].

## 2. Materials and Methods

### 2.1. Design

Within the IMPRESS project, we developed and evaluated a psychological support website for LTRs using the Person-Based Approach [8], which consisted of (1) planning, (2) design, (3) development, and (4) evaluation phases. In the planning phase, we identified the key issues, needs, and challenges the website must address. In the design phase, we established the main goals of the website and developed a prototype. In the development phase, feedback from a user panel was gained to make changes to the website. In the evaluation phase, we assessed the usability and added value of the website among LTRs using a pre-post design (see Figure 1).

### 2.2. Participants

For the development phase and evaluation phase, participants were recruited at one mental healthcare institute for those affected by cancer (Helen Dowling Institute), two academic medical centers (UMC Utrecht, Radboudumc), and through the social media channels of patient associations. Participants were considered eligible when diagnosed with advanced cancer with a confirmed response or long-term stable disease while on immunotherapy or targeted therapy. At least a third control scan after starting immunotherapy or targeted therapy should have confirmed the response to treatment. Participants needed to be adults, able to sufficiently use and understand the Dutch language and have internet access.

### 2.3. Procedures

#### 2.3.1. Planning

First, we examined the knowledge we gained from our earlier qualitative research and ecological momentary assessment studies in LTRs [1,12]. In our qualitative studies, we found that LTRs faced challenges in redefining their identity. They often felt caught between not identifying as patients yet not feeling entirely healthy, contributing to them going back and forth between hope and despair due to ongoing uncertainties. They were frequently confronted with an uncertain life perspective during medical check-ups. Upon realizing that returning to their pre-diagnosis life was impossible, they had to adjust to a new normal [1]. In response, LTRs are proactively seeking ways to regain control, shift their perspective, and realign their lives with value [12]. Exploratory findings from our Ecological Momentary Assessment study into the resilience shown in the daily lives of LTRs suggested that optimism, illness acceptance, mindfulness, and positive emotions, in general, are supportive factors to help LTRs manage stressors in daily life [13].

To elicit LTRs’ views on what key issues the website needs to address, we participated in the volunteer day of the patient association Longkanker Nederland. A brainstorming session with volunteers (including LTRs and their close others) highlighted peer support, communication between LTRs and their close others, and work-related issues as key topics. Despite their best efforts, close others often struggled to find the best ways to support LTRs and take care of themselves. Additionally, the accessibility of the website emerged as a crucial topic, emphasizing the need to provide LTRs with information through various formats (e.g., text, video, or animation). Lastly, we also consulted psychologists who have specific experience with treating metastatic cancer patients responding to immunotherapy or targeted therapy and other (international) researchers in the field of psycho-oncology to help us identify the issues and needs the website should address.

We presented all input to the scientific advisory board of the IMPRESS project, which comprised a patient (*n* = 1), patient representatives (*n* = 2), an oncologist (*n* = 1), researchers (*n* = 2), and researchers who also worked as psychologist (*n* = 3) in the field of psycho-oncology. The main topics that emerged during the group discussion included living with uncertainty, closeness to others, mourning one’s losses, acknowledging death and dying, and adapting to life with cancer.

#### 2.3.2. Website Design

**Figure 2:**
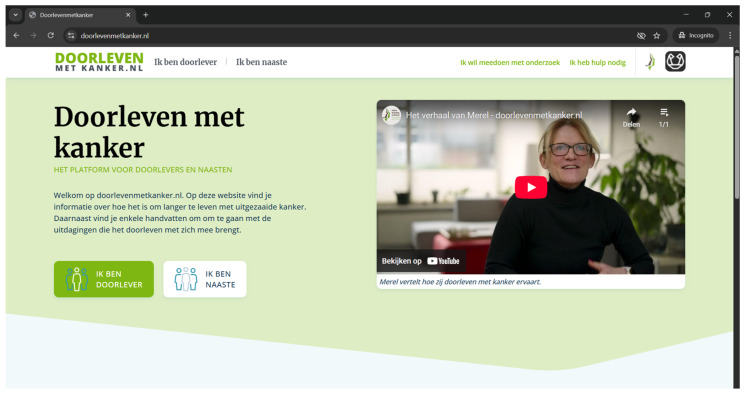
Homepage of the website.

Together with the scientific advisory board, we created the main goals of the website: (1) acknowledging difficulties and challenges that LTRs have to live with; (2) normalizing feelings induced by these difficulties and challenges; and (3) providing LTRs with tailored information and practical tools. We identified the primary objectives for the intervention design, which are based on the key issues highlighted in phase 1: living with uncertainty, relationships with close others, mourning losses, and adapting to life with cancer. Next, we outlined the essential features of the website required to meet these objectives. Specifically, we discussed and decided upon which themes should be covered on the website and the formats through which they should be presented (see Table 1). Together with a software provider, we created the design and structure of the website. With feedback from LTRs, the patient association Longkanker Nederland, and healthcare professionals, we created psycho-education texts. In collaboration with service providers and with feedback from healthcare professionals, we created therapist videos, LTR interview videos, and animation videos. We selected quotes describing how LTRs experience obtaining a long-term response from our previous interview studies [1,9]. Together with healthcare professionals, we developed exercises for the website. Lastly, we added links to other relevant websites. Eventually, this resulted in the prototype of the website (see Figure 2).

#### 2.3.3. Development

To test the prototype of the website, a user panel was assembled. The therapist, physician, or nurse practitioner identified eligible patients and provided them with relevant information. When patients were interested, the physician or research nurse informed the researcher, who then contacted them to explain the study procedure in more detail. After providing informed consent, participants completed a digital questionnaire via SurveyMonkey to provide sociodemographic and clinical characteristics. During the initial use of the prototype, an online think-aloud interview was conducted to gather concrete feedback for website improvement. Participants shared their screens, the online meetings were recorded, and notes were taken by the interviewer. Participants were instructed to verbalize their thoughts regarding the website’s appearance, usability, and content. No specific guidance was provided on which elements to examine, allowing participants to navigate and evaluate the website by themselves. A comprehensive list of feedback was compiled, leading to website adjustments. After two weeks of prototype use, feedback interviews on participants’ overall impressions of the website and their experiences with its content were conducted via telephone or online using Microsoft Teams. Key areas of discussion included appealing aspects of the website, suggestions for improvement, the perceived usefulness of the information provided and tools, and whether participants were utilizing these resources. The feedback from these interviews was used to further refine the prototype, and participants were consulted again until no significant improvements were needed. Additionally, a therapist and a nurse practitioner reviewed the prototype and provided feedback during an interview, which led to several improvements.

#### 2.3.4. Evaluation

For the evaluation of the website, the same recruitment procedures and criteria as those used during the development phase were applied. However, patients were not allowed to participate in both the development and evaluation phases. Upon providing written or digital informed consent, participants completed a set of baseline questionnaires via SurveyMonkey. They were then asked to use the website for one month. Six weeks after completing the baseline questionnaires, participants filled out follow-up questionnaires. In addition, a subsample of participants was interviewed about their experiences with the website.

### 2.4. Measures

One week before accessing the website (baseline) and six weeks after (follow-up), participants completed the Hospital Anxiety and Depression Scale (HADS) and the Brief Resilience Scale (BRS). We used these scales to describe the study population and to see if these variables were stable over time. We did not expect changes from baseline to follow-up.

Psychological distress was measured using the HADS [14]. It consists of 14 items examining anxiety and depression symptoms. An example item from the anxiety subscale is: “I feel tense”, with response options ranging from 0 (“Not at all”) to 3 (“Most of the time”). The HADS shows good psychometric properties in Dutch cancer patients, including reliability, validity, and internal consistency [15].

Resilience was measured with the BRS, which consisted of six items rated on a 5-point Likert scale (from 1 = totally disagree to 5 = totally agree), exploring the extent to which patients generally recovered quickly from adverse events [16]. An example item is the following: “I tend to bounce back quickly after hard times.” The Dutch version of the BRS showed good psychometric properties [17].

Usability (i.e., the ease with which users can effectively and efficiently interact with the website to achieve their goals) was assessed via the System Usability Scale (SUS) [18], which is often used to assess the usability of websites and online interventions, and categorizes them as either poor, okay, good, or excellent. This scale consisted of 10 items, such as “I think that I would like to use this system frequently.” This is rated on a 5-point Likert scale (from 1 = totally disagree to 5 = totally agree). The Dutch version of the SUS is a reliable and valid tool for assessing the usability of healthcare innovations [19].

Three open-ended questions assessed the feasibility of the website. We asked what participants found most appealing, what they missed, and whether and why they would (or would not) recommend the website to other LTRs.

Two research assistants conducted semi-structured interviews to gather additional information about the website’s usability and feasibility (see Table 2 for the topic guide). Both participants who were positive and negative about the website, as indicated by the questionnaires, were invited to the interview to gain the broadest possible insight into the website’s use and usefulness. The research assistants were not involved in the development of the website to avoid potential bias and ensure that the interviewees felt free to comment.

### 2.5. Analysis

#### 2.5.1. Power Analysis

We used a pre- and post-intervention design to evaluate the website. Following the recommendation of sample sizes of at least 30 participants in feasibility studies and a median sample size of 43 participants in publicly funded feasibility studies, we aimed to include at least 43 participants in our study [20].

#### 2.5.2. Quantitative Analysis

Paired-sample *t*-tests were used to examine whether patients’ psychological distress and resilience levels changed from baseline to six weeks after gaining access to the website. Participants with missing data were excluded from the analysis.

Descriptive statistics of the SUS provided an indication of how patients assessed the usage of the website. The website was considered feasible when the majority of participants rated the website as good or excellent.

#### 2.5.3. Qualitative Analysis

Data from the open-ended questions and semi-structured interviews were analyzed with the constant comparative method of the thematic analysis approach [21] using Atlas.ti (version 25) software. Data analysis started as soon as the follow-up questionnaires were completed, and the first interview was transcribed verbatim by the research assistants. Two researchers independently coded the open-ended questions and interviews and constantly compared coding schemes until they reached consensus. After all data were analyzed and consensus was reached, the research team grouped the codes referring to the same topic into subthemes and grouped the subthemes into themes.

## 3. Results

### 3.1. Development

#### 3.1.1. User Panel

Between May and July 2024, twelve patients were enrolled in the user panel. Three patients dropped out after receiving instructions due to illness progression (*n* = 1) and unknown reasons (*n* = 2). Ultimately, nine participants were included in the analysis, with ages ranging from 40 to 66 years (*M* = 56.00, *SD* = 7.42). Five participants were identified as women. All participants had at least an intermediate level of education, with four having attended university. Eight participants were diagnosed with metastatic lung cancer, and one participant was diagnosed with metastatic melanoma. The majority of participants were treated with immunotherapy (*n* = 6). The time since diagnosis varied from 25 to 88 months (*M* = 53.00, *SD* = 19.93). Two participants withdrew after the think-aloud interview due to adversities in their personal life. The data retrieved from the think-aloud interviews of these participants were used in the analysis.

The user panel was completed with a healthcare psychologist and nurse practitioner (author J.v.d.S). In June 2024, they also reviewed the website and provided feedback. The healthcare psychologist worked at the Helen Dowling Institute and had expertise in psychological treatment for cancer patients and, in particular, LTRs for four years. The nurse practitioner worked for 10 years at the Department of Respiratory Diseases, University Medical Centre Utrecht, with extensive experience in treating LTRs.

#### 3.1.2. Feedback on Design and Usability

While some participants felt that the website’s design was somewhat impersonal, for example, because the colors (i.e., different shades of blue and green) reminded them of the clinical environment of hospitals, the majority believed that the calm design was appealing and fitting for the website. Having numerous headings above the text helped participants understand what information they could find on the page.

A key area for improvement regarding design and usability was the excessive click-through options, which caused some participants to lose track of their progress. They reported not always knowing which pages they had already viewed. Consequently, we decreased the number of click-through options and highlighted the current page in the menu bar. Some pages initially appeared too similar, so we ensured the differences were more noticeable, for example, by incorporating different images on each page.

#### 3.1.3. Feedback on Feasibility

In terms of content, the conversation aid was helpful for users as it highlighted the significance of communication with those around them and encouraged deeper contemplation of the themes presented on the website. Presenting experiences from other LTRs through videos and quotes throughout the website significantly distinguished it from other websites, according to the user panel. Participants particularly appreciated the videos and quotes for their strong sense of recognition. The user panel specifically noted that the page on actively taking a role in your own medical care was very helpful as it provided LTRs with a sense of control amidst the uncertainty.

#### 3.1.4. Prototype Modification

Based on the feedback, several content modifications were made to improve the website. First of all, it was recommended to avoid providing information that could vary significantly for each LTR. For example, the text “At first, you may have a check-up scan every month. Later, this may be every 3 months, 6 months, or a year” was suggested to be removed and state that the time between scans may vary during the disease process. Secondly, it was noted that technical terms should be used with caution. For example, the term “illness progression” caused confusion among participants, as “progression” typically implies “positive advancement” in daily life. This was replaced by “illness worsening”. Thirdly, participants expressed a desire for specific information on, for example, supporting one’s children, nutrition and exercise, dealing with work, and psychosocial support and care. Consequently, we added references to specific websites providing information on these topics.

### 3.2. Evaluation

#### 3.2.1. Study Sample

From September to November 2024, 53 patients joined the evaluation panel. In total, 10 participants withdrew for the following reasons: a lack of access to the website (*n* = 4), disease worsening (*n* = 3), a lack of connection to the study (*n* = 1), technical errors preventing the completion of the follow-up questionnaire (*n* = 1), or unspecified reasons (*n* = 1).

Ultimately, 43 participants were included in the quantitative analysis, and 15 participants were included in the qualitative analysis. The 43 participants were aged from 24 to 73 years (*M* = 57.23, *SD* = 8.75), and the majority identified as women (*n* = 29, 67.44%). Most participants were diagnosed with lung cancer (*n* = 25, 58.14%) and treated with immunotherapy (*n* = 25). Time since diagnosis varied from 6 to 168 months (*M* = 45.56, *SD* = 32.70). Table 3 provides the sociodemographic and clinical characteristics of the participants. 

#### 3.2.2. Psychological Distress and Resilience

In line with our expectations, the results indicated that psychological distress did not change from baseline (*M* = 13.42, *SD* = 6.85) to follow-up (*M* = 13.58, *SD* = 5.92). The same applied to the ability to manage adversities, which also did not change from baseline (*M* = 3.66, *SD* = 0.56) to follow-up (*M* = 3.50, *SD* = 0.63).

#### 3.2.3. Design and Usability

One participant did not fully complete the SUS and was, therefore, excluded from the analysis. The average score on the SUS was 70.38. Of the 42 participants, 10 (23.8%) rated the website’s usability as “excellent”, 18 (42.9%) rated the website as “good”, 10 (23.8%) rated the website as “moderate”, and 4 (9.5%) rated the website as “poor”.

Qualitative analysis of open-ended questions and semi-structured interviews also revealed that participants’ opinions on usability were divided in terms of design, user-friendliness, and tone (see Table 4 for an overview of themes and quotes). Regarding design, some participants mentioned that they found the use of colors and illustrations calm and appropriate for the topic, while a few expressed a desire for more color and found the illustrations somewhat childish. User-friendliness was supported by the clear organization of the relevant main themes, although the numerous click-through options sometimes caused participants to lose track of where they were. In terms of the use of language, most participants indicated that the tone and language used on the website were accessible, concise, and personal, while some noted that it occasionally came across as impersonal and directive.

#### 3.2.4. Feasibility

Qualitative analysis of the open-ended questions and semi-structured interviews revealed four main themes regarding the feasibility of the website that were similar to the guiding principles of the website: (1) acknowledgment and normalization; (2) tailored information; (3) tools; and (4) recommendations to fellow LTRs. Please see Table 4 for themes, subthemes, and quotes.

***Acknowledgment and normalization.*** Participants experienced a strong sense of recognition with themes such as feeling healthy and ill and ongoing uncertainty. Peer experiences, for example, in the form of quotes and portrait videos, made participants feel less alone.

Several areas for improvement regarding recognition and normalization were mentioned by the evaluation panel. For example, in response to the information about the importance of monitoring one’s body, participants desired more acknowledgment. They reported that, due to various physical complaints, it can sometimes be very difficult to monitor your body or notice any changes occurring. Additionally, despite the added referrals as a result of feedback from the user panel, recognition was lacking for the emotions and challenges experienced by LTRs with (young) children. Although participants appreciated the availability of some information, specifically for their close others on the website, they indicated that much more information would be valued.

***Tailored information.*** The referrals, references to research, texts on relevant themes, and animation videos provided the evaluation panel with tailored and relevant information. They noted that the website, through its referrals, had become a repository of information pertinent to LTRs and could be seen as a guide for LTRs. The references to research allowed participants to understand the basis of the website’s information. Specifically, participants mentioned that texts on the psychological impact of a long-term response to treatment, the idea that making practical arrangements about end-of-life care can actually create space to continue living, and the considerations around the ability or desire to work were particularly informative. The animation videos were helpful in conveying information concisely and prompted participants to reflect on their own situation.

Participants noted that certain information could sometimes seem confronting. For example, some participants with an optimistic outlook or those who were doing well preferred not to read about the potential challenges and end-of-life issues. They expressed a desire for information that focused more on living. Additionally, participants indicated a need for more specific information, such as details on medical treatments, sexuality, work, and insurance, information specifically for young LTRs, when and how a psychologist can help, and managing relationships with children. Furthermore, the need for information specifically aimed at close others was again emphasized.

***Tools.*** The website features various practical tools, such as conversation aids, mindfulness exercises, the value task, and tips from psychologists or psychiatrists, which participants greatly appreciated and were eager to use. The existence of the website itself was also seen as supportive, as it provided hope that there were increasing numbers of LTRs living long lives, which helped broaden their perspective and foster an optimistic outlook. LTRs also wanted to use the website to inform their close others about their complex situation. The relevant themes offered LTRs input for reflection or discussion. Some participants mentioned that the website helped them face their reality, whereas they had previously tended to downplay the severity of their situation.

Participants missed the opportunity to interact on the website, such as a forum. They expressed a desire to use the website to connect with fellow LTRs. Additionally, they mentioned that it would be helpful to have a chat function, allowing them to ask questions to various healthcare providers.

***Recommending the website to fellow LTRs.*** In total, 38 participants (88.37%) noted that they would recommend the website to other LTRs. Out of 43 participants, 5 (11.63%) responded with “no comment” or “not applicable”. Five participants (11.63%) cautiously recommended the website, citing concerns that the website might unsettle users by addressing themes (e.g., end-of-life) that users are currently not focused on. The majority, 33 participants (76.74%), stated they would highly recommend the website to other LTRs, highlighting the wealth of information and practical tips that could serve as a guide amidst uncertainty.

Some participants suggested that the website should be made available soon after diagnosis, especially when there is a chance that the patient may become an LTR because the website can serve as a valuable guide through their disease process. Moreover, participants who were LTRs for many years indicated that they had already discovered how to manage these challenges by themselves and did not find the website particularly useful. Yet, others stated that the website could be appropriate at various times, as LTRs engage with different themes at different stages, allowing them to seek out information on the website that meets their needs at any given moment.

#### 3.2.5. Final Adaptations

Based on the comprehensive evaluation, we discussed within the research team which adaptations needed to be made before we officially launched the website. To improve usability, the number of clickable options was further reduced, and various topics were organized into colored sections to enhance the website’s clarity and ease of navigation. All videos on the website were subtitled to improve accessibility. We decided not to provide additional information, for example, about dealing with children, as the website already referred to a center with expertise in this area, and the creators of the website lacked this specific knowledge. Due to privacy legislation and the unavailability of moderators for the website, it was decided not to implement a forum or chat function. After these final adjustments and decisions, the website was officially launched in March 2025.

## 4. Discussion

The aim of this study was to develop and evaluate an online website with evidence-based information for advanced cancer patients who have obtained a durable response to immunotherapy or targeted therapy in co-creation with patients, psychologists, and service providers according to the Person-Based Approach [11]. Following the planning, design, and development phases, the website underwent a positive evaluation. Participants’ levels of psychological distress and resilience remained stable while using the website. The usability of the website was evaluated as good, and its content was deemed of additional value. The majority of participants indicated they would recommend the website to other LTRs.

As expected, we found no effects of using the website on psychological distress and resilience. This is in line with a meta-analysis, including 23 studies, which found no effect of web-based interventions for advanced cancer patients on anxiety or depression symptoms [22]. They reported that this was possibly due to a lack of personal informatics (i.e., tools that help people collect personally relevant information) [22,23]. Another potential explanation might be the relatively short time frame between website usage and completing the questionnaire. While the website provides various tools aimed at enhancing coping strategies, LTRs need to practice and incorporate these strategies into their daily lives. Although initial learning may take place during website use, applying these techniques may take longer [24]. Furthermore, the lack of psychosocial guidance for website users may hinder their ability to effectively integrate the strategies provided into their daily lives. Research indicates that unguided online interventions are often less effective compared to those that include guidance [25].

In terms of usability, the majority of participants rated the website as good. On average, websites or digital interventions have a similar rating (i.e., an average score of 68 on the System Usability Scale) [26]. For example, research on the creation of a website tailored for end-of-life planning for advanced cancer patients [27] and a study on a recovery application designed for lung cancer patients [10] received a comparable evaluation.

The website was developed based on the principles of acknowledging the challenges faced by LTRs, normalizing their feelings, and providing tailored information and practical tools. Our evaluation indicated that the website acknowledged the psychological impact of living long-term with cancer for LTRs, validating both negative and positive emotions that come with a long-term response, as described in earlier research [1,28]. Additionally, our study highlighted the need to acknowledge the challenges and burdens experienced by close others, confirming findings from a qualitative study showing that partners tend to sacrifice their own needs, impacting their wellbeing and increasing their anxiety [29].

Regarding tailored information, our study showed that LTRs experience ambivalent emotions towards information about end-of-life care and preparation on the website. Similarly to the literature, some LTRs preferred to focus on life instead of the end of life, while others recognized that making arrangements in advance could be helpful [12,30]. Previous studies showed that talking about the end of life improved cancer patients’ quality of life by helping them understand their prognosis and treatment options and, in turn, reduced uncertainty and fear [31]. This underscores the importance of discussing end-of-life matters and ensuring they are handled with sensitivity and care.

Our study demonstrated that LTRs use the website as a tool to explain their complex situations to close others. This aligns with research indicating that LTRs often feel misunderstood [1]. Friends and family often struggle to understand LTRs’ conditions, partly due to the misconception that cancer is either curable or terminal. The fact that LTRs do not always look ill exacerbates these challenges [2]. In addition, the attention of close others often decreases as LTRs live longer, while they still feel a great need for social support [5]. Showing the website to close others helped LTRs explain the challenges they faced were common for their situation, hopefully increasing understanding and support.

### 4.1. Strengths and Limitations

Our study demonstrates several strengths. Most notably, the website is grounded in extensive prior research conducted by the authors on LTRs [1,12]. Additionally, the website was developed by a multidisciplinary team comprising patients, relatives, patient representatives, therapists, oncologists, and nurse practitioners. This collaborative approach ensured that researchers had a clear understanding of the wishes and needs of LTRs throughout the entire research and development process.

Our results should also be viewed in light of some limitations. Initially, the researchers planned to analyze (1) technical data during the website evaluation, such as the frequency of website visits, button clicks, and video views, and (2) data from a custom-made questionnaire about the extent to which certain topics were adequately covered and tools were offered to help manage related challenges. However, due to incorrect technical settings, the amount of technical data that could be retrieved was limited and unreliable. Moreover, due to programming errors, the quantitative data of the custom-made questionnaire could not be interpreted. As a result, conclusions about the website’s usability had to rely solely on the System Usability Scale scores and qualitative data.

### 4.2. Implications for Clinical Practice and Research

During brainstorming sessions, feedback discussions with LTRs and therapists, and the evaluation of the website, it became clear that LTRs hoped that the website could facilitate contact with other members. As noted, this feature was not included in the website due to complications with privacy legislation and security. For example, research highlighted the importance of active moderators in creating a secure and supportive environment for individuals to share their personal experiences [9]. This contrasted with our aim to ensure that the website remained accessible and sustainable, requiring minimal manpower to reduce the pressure on healthcare. Nevertheless, the needs of LTRs cannot be ignored, and it is necessary to facilitate more peer contact among LTRs.

The section of the website designed for LTRs is grounded in thorough research. In contrast to this, a significant gap in research remains regarding the coping mechanisms and challenges faced by the close others of LTRs. In the first phase of our study, it already became clear that close others would also need tailored information and support. Conducting further research, such as an interview study, could offer deeper insights and serve as a foundation for refining the section of the website tailored to close others to better align with their wishes and needs.

In the future, there are still challenges in the implementation of the website. It is important to keep healthcare providers, patient associations, and other initiatives informed about the existence of the website. Regularly adding new content may help with this, ensuring that visitors continue to return to the website.

## 5. Conclusions

Due to new treatment options, the group of patients who live longer with cancer is rapidly growing [7]. While this is promising, many LTRs find it difficult to move forward with their lives after obtaining a long-term response [1,6,12]. To address these challenges, we developed the Dutch website www.doorlevenmetkanker.nl in collaboration with LTRs, healthcare professionals, researchers, and service providers. We identified the key issues (i.e., living with uncertainty, relationships with close others, mourning losses, and adapting to life with cancer) and established the website’s main goals: acknowledging and normalizing the emotions, difficulties, and challenges LTRs face and providing tailored information and practical tools. Through repeated feedback from a user panel, the prototype was further optimized. The usability was largely rated as good to excellent. Interview data indicated that participants experienced recognition through portrait videos and quotes, valued the psycho-education provided via written text and (animated) videos, and made use of the practical tools (e.g., conversation aid and mindfulness exercise), confirming that the main goals of the website were achieved. Following this positive evaluation and some final adjustments, the website was officially launched in March 2025 in the Netherlands.

## Figures and Tables

**Table 1:** Overview of website features.

Website Page	PE ^1^ Text	PE ^1^ Therapist Video	LTR Interview Video	Animation Video	Quotes	Exercises	Referral
1. Long-term responders							
1.1. When everything is uncertain…							
1.1.1. Living with uncertainty	X		X	X	X		
1.1.2. Fear that the disease will get worse	X	X			X	Mindfulness Conversation aid	
1.1.3. Monitoring your body	X				X		
1.1.4. Your role in your care	X				X		X
1.2. The people surrounding you							
1.2.1. Talking about your disease	X	X	X		X		X
1.2.2. Changes in daily life	X				X	Conversation aid	
1.2.3. Contact with peers	X				X		X
1.3. Your old life and the end of life							
1.3.1. Saying goodbye to your old life	X				X	Conversation aid	
1.3.2. Thinking about the end of life	X			X	X	Conversation aid	
1.4. Getting back on track							
1.4.1. Making changes	X		X		X		
1.4.2. What matters to you	X	X			X	Value task	
1.4.3. Cancer and work	X				X		X
2. Close others							
2.1. Living long-term with cancer	X			X			
2.2. Supporting a long-term responder	X						
2.3. Taking care of yourself	X						
3. I want to participate in research	X				X		
4. I need help	X						X

^1^ PE = psycho-education.

**Table 2:** Topic guide for semi-structured interviews.

Topic	Questions
Design	How did you experience the design of the website? Did it appeal to you, or not? How did the design of the website make you feel?
Usability	What do you think of the website’s navigation? How easy or difficult was the use of the website? How easily could you find information or content on the website? What do you think of the language used on the website? Was it appealing, or not?
Feasibility	How did the content of the website make you feel? What page of the website was most appealing to you? What page of the website did you like the least? What would you change about the website? What did you miss on the website?
Consequences	Did using the website help you? In what way? Have you been thinking, feeling or doing things differently after using the website? To what extent did you feel supported by using the website? Why? Would you visit the website again in the future? What for?
Recommendation	Would you recommend the website to other LTRs? Why? Thinking back on your own diagnosis and treatment, what do you think is the best moment to visit this website? Why? Would you recommend your close others to visit the website?
Closing	Is there anything else you would like to discuss?
	What was it like taking part in this interview?
	Do you have any questions left about this study?

**Table 3:** Clinical and sociodemographic characteristics of the evaluation panel.

	*n*	(%)
Age, mean (SD)	57.23	8.75
Gender		
*Male*	14	32.56
*Female*	29	67.44
Educational level		
*Practical*	1	2.33
*Intermediate*	20	46.51
*Theoretical*	22	51.16
Working status		
*Employed/Student*	6	19.95
*Disabled*	21	48.84
*Sick*	8	18.61
*Retired*	4	9.30
*Volunteering*	4	9.30
Relationship status		
*Single*	3	6.98
*Married/Living together*	36	83.72
*Divorced*	2	4.65
*Widowed*	2	4.65
Severely distressed (HADS ≥15)	16	37.21
Cancer type		
*Lung cancer*	25	58.14
*Melanoma*	9	20.93
*Breast cancer*	7	16.28
*Esophageal cancer*	1	2.33
*Cervical cancer*	1	2.33
Time since diagnosis in months, mean (SD)	45.56	32.70
Treatment		
*Immunotherapy*	25	58.14
*Targeted Therapy*	18	41.86

Note: SD = standard deviation.

**Table 4:** Themes and subthemes divided into positive feedback and suggestions for improvement, and quotes regarding the usability and feasibility of the website.

Themes and Subthemes on Positive Feedback	Quotes	Subthemes on Suggestions for Improvement	Quotes
**1. Usability**			
Calm colors and appropriate illustrations	*“I think the website looks appealing. The green color gives it a natural look. It also feels like the continuation of life. It’s not overly somber; it is fresh like spring.”* -woman (52), melanoma	Little use of color and childish illustrations	*“I found the illustrations a bit childish. They’re clear and well-organized, but yeah... A lot of people are going to use it [the website]. They have all kinds of educational levels of course, so it has to be understandable for everyone, but because of that I sometimes found it a bit simple.”* -woman (55), lung cancer
Clear main themes	*“It’s pretty clear, isn’t it? There are 4 clear chapters that you can go to.”* -man (53), melanoma	Losing track due to many click options	*“I find it challenging to stay on track. The website contains a lot of information and numerous referrals, making it hard to maintain an overview. I often wonder: Where am I now? How do I get back? Should I continue with a related subtopic?”* -woman (55), lung cancer
Accessible, concise, and personal in language/tone	*“The tone of voice of the website felt warm and familiar.”* -man (57), lung cancer	Sometimes impersonal or too directive in language/tone	*“What bothered me a bit was: ‘are you struggling with this? then this is a solution.’ As if someone has the wisdom on how to deal with this. I prefer offering options or possibilities. Yeah, well, I don’t think this happened often, but every now and then I came across something like this.”* -woman (55), lung cancer
**2. Acknowledgement and Normalization**			
Attention for LTRs	*“What I found most appealing is the attempt to get close to the patients.”* -man (63), lung cancer	Limited attention paid to close others	*“Just as there are all kinds of videos for the patients, they should also be there for their close others. They also want recognition and confirmation and perhaps a little encouragement like ‘hey, we see you’. Yes, because they really have it just as hard, don’t they? Because they have to do all that extra stuff and watch someone else being really sick, which causes a lot of sadness. Plus, 80% of the people ask them: hey, how is the patient? That’s pretty lonely. So it would be really cool if you had a website that says: hey, how are you?”* -woman (49), lung cancer
Acknowledging difficulties	*“Wow… finally, someone describes exactly how I feel!”* -woman (59), lung cancer	Not acknowledging difficulties with checking your body	*“On the page ‘checking your body’ there is actually nothing acknowledging, for example that it is really complicated to analyze how your body feels. So, you want someone to analyze how the body feels, but how? It is a complicated thing, isn’t it?”* -woman (54), lung cancer
Finding recognition in peer experiences	*“I was especially drawn to the stories of peers. It’s comforting to realize that we form a unique group.”* -man (53), melanoma		
Co-creation with LTRs	*“The website connects with people who are sick and are sentenced to death again and again and still live, just like me... You can simply see that the website was built by such people.”* -man (46), melanoma		
The term “LTR” provides hope	*“The word ‘doorleven’ (literally: continue living) has the most impact on me. You are not dead, you are going to live with cancer.”* -man (63), lung cancer		
**3. Tailored Information**			
Specialized website referrals	*“The explanations are clear, particularly the references to sites that discuss the topics in more depth, preventing the need to reinvent the wheel.”* -woman (49), breast cancer	Lack of focus on living	*“In addition to the motto ‘be always prepared,’ I also embrace the saying ‘So far, so good!’ I feel that this positive perspective is somewhat missing from the site. An LTR continues their life, and that’s wonderful!”* -man (52), melanoma
Research references	*“The scientific research is very valuable. On the website you can read what happens behind the scenes. I do not search the internet that much, because you often end up on things that do not apply to you. Now, I have a direct site that is reliable and that is about you and fellow patients.”* -man (66), melanoma	Lack of specific information	*“I am ill and have young children… How do other young mothers manage parenting while dealing with illness, especially when it comes to the thought of leaving their children behind?”* -woman (39), lung cancer
Clear psycho-education	“*The website does make it clear that it [living with advanced cancer] has a huge impact on people, and I think that’s good. I’m a bit more light-hearted by nature, but I think it’s good that it’s clear here, because it can also end badly. I think the texts are good.”* -woman (62), lung cancer		
Accessible animated videos	*“I found the videos useful and easy to follow, because they are in animation form and don’t take long to watch. The movies are nice, short, and concise, in which the important things are touched upon.”* -woman (62), breast cancer		
**4. Tools**			
Provided tools	*“The questions of the conversation aid were most appealing to me. It’s nice to have these questions and to think about them.”* -woman (24), cervical cancer	Lack of interaction	*“I miss the opportunity to interact and respond. Having that feature would motivate me to visit the website repeatedly.” -*man (62), lung cancer
Inform close others	*“I really came across things on the website ‘yes that is exactly what I feel, that is exactly what I also come across’. I immediately thought something like I have to tell my father and my husband, and my close others to read that website, and you should not go to the page for loved ones, but for the LTR and read that.’”* -woman (39), lung cancer		
Providing hope	*“I would probably offer the website a few months after the diagnosis, because there are things on it that are nice to read. You can see the website as something hopeful, because you can read that people often live longer.” -*woman (62), lung cancer		
Input for reflection and discussion	*“My close others also found it complicated: ‘You’re cured, aren’t you? Yeah, what’s your problem?’ Of course, that’s not how they react. But I think that’s what they think. Of course, that’s not true. Due to the conversation aid, I think it’s a good idea to have that conversation again with my family, my friends. We don’t have to give a daily health update anymore: am I dying yet? But it’s like: I’m not completely back yet or something. Cancer still plays a role.”* -woman (52), melanoma		
Sense of reality	*“I thought it was good that all those quotes were on there, no matter how heavy they are, because they are really from people who have cancer too. Look, if you deal with it the way I do, then ehm you might also have the tendency to make it a bit too small sometimes. But in this way I do have support from peers who are going through the same thing. So it was helpful to read those quotes.”* -man (53), melanoma		
**5. Recommendation to Other LTRs**			
Clear recommendation	*“I would definitely recommend the website. It is a beautiful website and very valuable. There is a lot of information and references to even more information that directly relate to LTRs. And if I speak for myself, there was a lot of recognition and I got practical tips that I plan to act on. Reading all this can be confronting at times. Sometimes you may not want to, but it becomes part of the new you.”* -man (66), melanoma	Cautious recommendation	*“It depends on the person and what they are doing. Many things I read were recognizable but did not provide any new insights. I feel that I have found a balance in life. However, by focusing on the illness or reading a lot on the site about topics that no longer concern me (for example, thinking about the end of life), that balance was sometimes disturbed. I don’t feel like dwelling on that. Sometimes, I even felt a bit irritated by the ready-made answers.”* -woman (55), lung cancer
Use shortly after diagnosis	*“I think the website is a very good user manual for those who are newly palliative. I have been palliative for a long time and have already figured everything out myself. I see this website as a guide.”* -woman (62), lung cancer		
Use at various times	*“There are of course different themes on the website, which are relevant at different times. For the moment just before a scan, there is a heading about how to deal with stress, so I would read that then. Furthermore, at times when I am busy with everything I still have to arrange for the last phase of my life, I would also look at the website.”* -woman (24), cervical cancer		

## Data Availability

A minimal dataset is available in the Supplementary Material. The qualitative data presented in this study are available upon request from the corresponding author due to the privacy of participants’ information.

## Supplementary Materials

The following supporting information can be downloaded at: https://www.mdpi.com/article/10.3390/curroncol32050284/s1.

## Abbreviations

The following abbreviations are used in this manuscript:

LTRs	Long-term responders
PE	Psycho-education
HADS	Hospital Anxiety Depression Scale
BRS	Brief Resilience Scale
SUS	System Usability Scale

## References

[1] Zwanenburg L.C., Suijkerbuijk K.P.M., van Dongen S.I., Koldenhof J.J., van Roozendaal A.S., van der Lee M.L., Schellekens M.P.J. (2022). Living in the twilight zone: A qualitative study on the experiences of patients with advanced cancer obtaining long-term response to immunotherapy or targeted therapy. J. Cancer Surviv..

[2] Kolsteren E.E.M., Deuning-Smit E., Chu A.K., van der Hoeven Y.C.W., Prins J.B., van der Graaf W.T.A., van Herpen C.M.L., van Oort I.M., Lebel S., Thewes B. (2022). Psychosocial Aspects of Living Long Term with Advanced Cancer and Ongoing Systemic Treatment: A Scoping Review. Cancers.

[3] Dunn J., Watson M., Aitken J.F., Hyde M.K. (2017). Systematic review of psychosocial outcomes for patients with advanced melanoma. Psycho-Oncology.

[4] Farland D.C. (2019). New lung cancer treatments (immunotherapy and targeted therapies) and their associations with depression and other psychological side effects as compared to chemotherapy. Gen. Hosp. Psychiatry.

[5] Lai-Kwon J., Heynemann S., Flore J., Dhillon H., Duffy M., Burke J., Briggs L., Leigh L., Mileshkin L., Solomon B. (2021). Living with and beyond metastatic non-small cell lung cancer: The survivorship experience for people treated with immunotherapy or targeted therapy. J. Cancer Surviv..

[6] Kamminga N.C., van der Veldt A.A., Joosen M.C., de Joode K., Joosse A., Grünhagen D.J., Nijsten T.E., Wakkee M., Lugtenberg M. (2022). Experiences of resuming life after immunotherapy and associated survivorship care needs: A qualitative study among patients with metastatic melanoma. Br. J. Dermatol..

[7] Haslam A., Gill J., Prasad V. (2020). Estimation of the percentage of US patients with cancer who are eligible for immune checkpoint inhibitor drugs. JAMA Netw. Open.

[8] Marshall-McKenna R., Kotronoulas G., Kokoroskos E., Gil Granados A., Papachristou P., Papachristou N., Collantes G., Petridis G., Billis A., Bamidis P.D. (2023). A multinational investigation of healthcare needs, preferences, and expectations in supportive cancer care: Co-creating the LifeChamps digital platform. J. Cancer Surviv..

[9] Timmerman J.G., Tönis T.M., Weering M.G.H.D.-V., Stuiver M.M., Wouters M.W.J.M., van Harten W.H., Hermens H.J., Vollenbroek-Hutten M.M.R. (2016). Co-creation of an ICT-supported cancer rehabilitation application for resected lung cancer survivors: Design and evaluation. BMC Health Serv. Res..

[10] Olsson M., Eliasson I., Kautsky S., Segerstad Y.H.A., Nilsson S. (2024). Co-creation of a digital platform for peer support in a community of adolescent and young adult patients during and after cancer. Eur. J. Oncol. Nurs..

[11] Yardley L., Morrison L., Bradbury K., Muller I. (2015). The Person-Based Approach to Intervention Development: Application to Digital Health-Related Behavior Change Interventions. J. Med. Internet Res..

[12] Zwanenburg L.C., van der Lee M.L., Koldenhof J.J., Suijkerbuijk K.P.M., Schellekens M.P.J. (2024). What patients with advanced cancer experience as helpful in navigating their life with a long-term response: A qualitative study. Support. Care Cancer.

[13] Zwanenburg L.C., van Roekel E., Suijkerbuijk K.P.M., Koldenhof J.J., Schuurbiers-Siebers O.C.J., van der Stap J., van der Lee M.L., Schellekens M.P.J. (2025). Resilience in advanced cancer patients who obtain a long-term response to immunotherapy or targeted therapy: An Ecological Momentary Assessment study. Ann. Beh. Med..

[14] Zigmond A.S., Snaith R.P. (1983). The hospital anxiety and depression scale. Acta Psychiatr. Scand..

[15] Spinhoven P., Ormel J., Sloekers P.P.A., Kempen G.I.J.M., Speckens A.E.M., Van Hemert A.M. (1997). A validation study of the Hospital Anxiety and Depression Scale (HADS) in different groups of Dutch subjects. Psychol. Med..

[16] Smith B.W., Dalen J., Wiggins K., Tooley E., Christopher P., Bernard J. (2008). The brief resilience scale: Assessing the ability to bounce back. Int. J. Behav. Med..

[17] Leontjevas R., de Beek W., Lataster J., Jacobs N. (2014). Brief Resilience Scale-Dutch Version [database record]. APA PsycTests.

[18] Bangor A., Kortum P.T., Miller J.T. (2008). An Empirical Evaluation of the System Usability Scale. Int. J. Hum. Comput. Interact..

[19] Ensink C.J., Keijsers N.L.W., Groen B.E. (2024). Translation and validation of the System Usability Scale to a Dutch version: D-SUS. Disabil. Rehabil..

[20] Billingham S.A., Whitehead A.L., Julious S.A. (2013). An audit of sample sizes for pilot and feasibility trials being undertaken in the United Kingdom registered in the United Kingdom Clinical Research Network database. BMC Med. Res. Methodol..

[21] Braun V., Clarke V. (2022). Thematic Analysis: A Practical Guide.

[22] Kamalumpundi V., Saeidzadeh S., Chi N.-C., Nair R., Gilbertson-White S. (2022). The efficacy of web or mobile-based interventions to alleviate emotional symptoms in people with advanced cancer: A systematic review and meta-analysis. Support. Care Cancer.

[23] Hong Y.A., Hossain M.M., Chou W.S. (2020). Digital interventions to facilitate patient-provider communication in cancer care: A systematic review. Psycho-Oncology.

[24] Hamama-Raz Y., Pat-Horenczyk R., Perry S., Ziv Y., Bar-Levav R., Stemmer S.M. (2016). The Effectiveness of Group Intervention on Enhancing Cognitive Emotion Regulation Strategies in Breast Cancer Patients. Integr. Cancer Ther..

[25] van Helmondt S.J., van der Lee M.L., van Woezik R.A.M., Lodder P., de Vries J. (2020). No effect of CBT-based online self-help training to reduce fear of cancer recurrence: First results of the CAREST multicenter randomized controlled trial. Psycho-Oncology.

[26] Hyzy M., Bond R., Mulvenna M., Bai L., Dix A., Leigh S., Hunt S. (2022). System Usability Scale Benchmarking for Digital Health Apps: Meta-analysis. JMIR mHealth uHealth.

[27] Walsh C.A., Miller S.J., Smith C.B., Prigerson H.G., McFarland D., Yarborough S., Santos C.D.L., Thomas R., Czaja S.J., RoyChoudhury A. (2024). Acceptability and usability of the Planning Advance Care Together (PACT) website for improving patients’ engagement in advance care planning. PEC Innov..

[28] Burgers V.W.G., Bent M.J.v.D., Rietjens J.A.C., Roos D.C., Dickhout A., Franssen S.A., Noordoek M.J., van der Graaf W.T.A., Husson O. (2022). “Double awareness”—Adolescents and young adults coping with an uncertain or poor cancer prognosis: A qualitative study. Front. Psychol..

[29] Kolsteren E.E.M., Deuning-Smit E., Prins J.B., van der Graaf W.T.A., Kwakkenbos L., Custers J.A.E. (2024). Perspectives of patients, partners, primary and hospital-based health care professionals on living with advanced cancer and systemic treatment. J. Cancer Surviv..

[30] Arantzamendi M., García-Rueda N., Carvajal A., Robinson C.A. (2020). People With Advanced Cancer: The Process of Living Well With Awareness of Dying. Qual. Health Res..

[31] Hayashi Y., Sato K., Ogawa M., Taguchi Y., Wakayama H., Nishioka A., Nakamura C., Murota K., Sugimura A., Ando S. (2022). Association Among End-Of-Life Discussions, Cancer Patients’ Quality of Life at End of Life, and Bereaved Families’ Mental Health. Am. J. Hosp. Palliat. Med..

