# Supplementary material for: The Co-Creation of a Psychosocial Support Website for Advanced Cancer Patients Obtaining a Long-Term Response to Immunotherapy or Targeted Therapy
Source: Curr Oncol. 2025 May 19;32(5):284. doi: 10.3390/curroncol32050284 (PMC12110623; doi:10.3390/curroncol32050284)
Supplement: Supplementary file 1 [file curroncol-32-00284-s001.zip › curroncol-3622597-supplementary.pdf]

Minimal dataset belonging to *'The co-creation of a psychosocial support website for advanced cancer patients obtaining a long-term response to immunotherapy or targeted therapy.'*

Participants	HADS_baseline	BRS_baseline	HADS_followup	BRS_followup	SUS
1	33	3,166666667	26	2,333333333	87,5
2	9	4,166666667	11	4,166666667	75
3	6	4,666666667	3	4,333333333	37,5
4	22	2,166666667	18	2,666666667	75
5	14	4,166666667	15	3,333333333	95
6	12	3,666666667	22	2,666666667	72,5
7	12	4,666666667	16	4	90
8	9	4,5	8	4,5	67,5
9	16	4	15	3,5	67,5
10	9	4	8	3,666666667	72,5
11	36	4	26	2,666666667	52,5
12	17	3,333333333	16	3,833333333	77,5
13	11	3,833333333	10	4	75
14	18	3,166666667	20	3,5	
15	10	4	8	4,166666667	70
16	21	3,666666667	14	4,5	72,5
17	10	2,833333333	6	2,5	60
18	6	4,166666667	15	3,166666667	62,5
19	11	4,166666667	14	3,833333333	70
20	15	3	9	3,166666667	
21	20	3,333333333	20	3,333333333	80
22	5	4	13	3,833333333	72,5
23	19	3	15	2,333333333	77,5
24	14	2,833333333	17	3	60
25	17	3,166666667	18		90
26	3	4,166666667	3	3,666666667	55
27	11	2,833333333	18	3,166666667	77,5
28	8	4,5	11	4,333333333	85
29	3	4	6	4,5	65
30	12	3,5	12	3	
31	6	4	5	3,5	85
32	18	3,5	20	3,166666667	80
33	9	3,5	12	4	17,5
34	15	3,5	18	3,166666667	75
35	11	3,5	10	3	50
36	21	3,5	18	3,666666667	85
37	20	3,166666667	20	3,833333333	55
38	9	3	10	3,666666667	55
39	15	3,166666667	17	2,333333333	42,5
40	14	3,5	20	3,5	60
41	13	4,166666667	3	4,333333333	92,5
42	7	3,833333333	7	4,166666667	82,5
43	10	3,666666667	11	3,166666667	92,5
